# Dietetics

**Published:** 1876-04

**Authors:** 


### DIETETICS.

A man, simply because he is sick, is not to be starved, nor, on the other hand, can a man who is sick, as a rule, take such articles of food as a well man would be likely to take.

It may be doubtful whether a man when first taken sick should eat a large quantity of food, but one of the articles which he may have is *Indian gruel*, if not made too strong. If, however, you give permission that the patient may have gruel to take, unless you give special directions as to how it shall be made, you will very commonly find that the nurse has prepared a fair specimen of Indian pudding, and has been administering that for gruel.

In making Indian gruel there should be no more than a dessert or table-spoonful of the meal to a quart of water, and this should be boiled for a long time, keeping the quantity of water good throughout the entire boiling process.

Prepared in this manner, it may be made decidedly salt, and then administered to the patient during the first few days of his sickness. There is one article of diet which all persons may take under all conditions, and that is *milk*.

There are those who say they cannot take milk, that it makes them bilious, etc.; but that is not true. A person who is sick may take milk with the greatest possible advantage, because it contains, in a form easy of assimilation, all the elements essential for maintaining nutrition. It is the natural aliment of the young animal, and certainly answers a good purpose for the old animal, provided it is used properly. New milk, I do not hesitate to say, may be taken, as far as disease is concerned, in any and every condition. Perhaps it will require the addition of lime-water if marked acidity of the stomach is present; and perhaps a little gentian may be requisite to stimulate the stomach somewhat; and it may be necessary to give it in small quantities and repeat it often; but ice-cold milk can be put into a very irritable stomach, if given in small quantities and at short intervals, with the happiest effects. We have now come to believe, contrary to the teachings of our fathers, that *cold water*, even ice-cold water, is a most beneficial drink, and therefore permit our patients to have it as often as they wish, provided too much is not taken at any one time.

Now, *tea*, which is a wholesome beverage, and withal contributes somewhat to scandal, is very comforting, especially to a sick woman, and may be given without harm if it is sufficiently diluted with milk. When made very weak—just strong enough to give a flavor—well supplied with milk and perhaps a little sugar, it gives the patient a trifle of nourishment in a very palatable form.

There is another article which has long been known in the sick-room, and that is *beef-tea*. This is not only agreeable to the taste when properly made, but it is one of the very best methods of administering nourishment. There are many who say there is little or no nourishment in beef-tea, but it does nourish men, otherwise they could not live as long as they do upon it. I admit that bad beef-tea is not very nutricious; and it is perhaps the exception that patients get good beef-tea. It is this fact, perhaps, which, as much as anything, has led to its disuse. If, however, you will make beef-tea according to the directions I now give you, it will be found to be a most serviceable article among the dietetics of the sick-room:

Take a pound of the very best beef that can be obtained in the market,—the butcher will tell you that any kind of a piece answers to make beef-tea of, but that is not true,—cut it into small pieces the size of the end of the thumb, place it in a pint basin, cover with cold water, and then place the dish upon the back part of the range or stove, where the water will gradually get warmer and warmer, but will not reach the boiling point. Let it stand and simmer in this manner two hours. Then bring it forward, and boil over a quick fire twenty minutes, and immediately after pour the fluid from the beef, at the same time allowing the little particles which become detached to flow off with it. Now, if there is any fat in the tea, it is well that it should be removed, for the reason that the bile and pancreatic secretion may be unable to emulcify it, and it may do more harm than good. If you wish to be very precise upon this point, the tea can be set aside, and when perfectly cold all the fat can be removed from the surface in a flake; or the fat may be taken up by dropping a piece of flannel upon it as it floats on the surface of the warm tea.

It is not a good plan to strain the liquor, because this process will remove more or less of the little particles of beef, which are very essential to the value of the tea. It may now be salted and given hot or cold, as the patient may wish; and it may be given as soon as the pulse indicates any diminution in the force of the heart's action. What becomes of this article of diet when taken into the stomach? The advocates of the worthlessness and non-essential in beef-tea would answer that it makes but litile difference. I believe, however, that it is mostly taken up by the gastric veins, and at all events that it is exceedingly palatable and nutritious, and does do something more than simply warm the stomach and make the patient happy for a short time.

In case the patient's stomach is very irritable, so that large quantities of any substance cannot be borne, you may resort to *beef-extract* for nourishment.

The proper method of making this article is to take a pound of the best beef, cut it into small pieces and place it in a good-sized open-mouthed bottle; a pickle-jar is perhaps as convenient as any. Cork the bottle loosely, and then set it into a kettle of water, which is to be kept boiling for two hours. If the bottle is now removed it will be found to contain a considerable quantity of fluid, which may be turned off, the beef subjected to slight pressure to remove still more.

In this fluid we have a concentrated article of nourishment, and it may be given, after it has been seasoned, either pure or diluted, according to the condition of the stomach. Beef-extract is not nearly so palatable an article of food as rich beef-tea made in the manner described. Ordinarily, however, the tea is badly made, and contains but little beef and considerable water.—*Boston Journal of Chemistry.*

